# The influence of problem drinking persons on the quality of life of their surroundings depending on gender and age

**DOI:** 10.1192/j.eurpsy.2023.615

**Published:** 2023-07-19

**Authors:** V. Kuzminov, I. Linskiy, V. Zadorozhny, R. Lakinskiy, T. Tkachenko, O. Minko

**Affiliations:** Urgent psychiatry and Narcology, SI Institute of Neurology Psychiatry and Narcology NAMS of Ukraine, Kharkiv, Ukraine

## Abstract

**Introduction:**

The social consequences of alcohol affect not only drinkers, but also the people communicate with him. They are including: mental health, quality of life, health, living conditions and needs in using health system resources.

**Objectives:**

1,531 people were examined, who belonged to three qualitatively different comparison groups: patients with alcohol addiction (329 people); healthy relatives of patients with alcohol addiction (238 persons) and representatives of the general population (964 persons).

**Methods:**

clinical, clinical and psychopathological, methods of quantified scales and mathematical statistics.

**Results:**

The data obtained indicate that the majority of respondents who caused some harm to the respondents were men. But the level of harm of alcohol abuse of women was significantly higher. Alcohol abuse problems of microsocial environment (substance abuse, employment, financial problems, and health effects) had a more significant effect on depressive disorders in the control group than in the alcohol addiction group and their relatives. Significant correlation of depressive disorders due to the drunkenness of others was associated with fear for children, the possibility of aggression. Depressive disorders in the group relatives of patients with alcohol addiction correlated with aggression towards them with patients with alcohol addiction or persons in a state of alcohol intoxication, discomfort due to being with their relatives in public, at parties, inability to control alcohol use. It was shown that the presence of drinkers in the company of women significantly increases the specific weight of people with depressive disorders among them, while such an effect was not found among male respondents. It has been established that in the case of the presence of drinkers in the environment, the expressiveness of such manifestations of depression as insomnia increases the most in women; weight loss, sexual disorders; suicidal tendencies and feelings of guilt. The influence of presence of drinkers in the environment of children on tfrequency of manifestations of their discomfort is described. It has been confirmed that the most frequent cause of children’s ill health is their drinking parents, as well as other (besides parents and siblings) drinking relatives. It was found that in healthy respondents, the unfavorable features of the behavior of relatives and close drinkers are always directly correlated with indicators of the ill health of children in their environment, while in respondents addicted to alcohol, these correlations have a complex, mosaic nature.

**Conclusions:**

The study found the prevalence of depressive disorders in the microsocial environment of drinkers. A significant decrease in the quality of life was noted primarily in children and women who had drinking relatives with a large number of drunk days.

**Disclosure of Interest:**

None Declared

